# Correlation Analysis of Macular Function and Peripapillary Retinal Nerve Fiber Layer Thickness Following Successful Rhegmatogenous Retinal Detachment Surgery

**DOI:** 10.3390/biomedicines13040943

**Published:** 2025-04-11

**Authors:** María D. Díaz-Barreda, Ana Boned-Murillo, Isabel Bartolomé-Sesé, María Sopeña-Pinilla, Elvira Orduna-Hospital, Guisela Fernández-Espinosa, Isabel Pinilla

**Affiliations:** 1 Department of Ophthalmology, Obispo Polanco Hospital, 44002 Teruel, Spain; lodiba92@gmail.com; 2 Aragón Health Research Institute (IIS Aragón), 50009 Zaragoza, Spain; anabomu@hotmail.com (A.B.-M.); issbartolome@gmail.com (I.B.-S.); mariasopenapinilla@gmail.com (M.S.-P.); eordunahospital@unizar.es (E.O.-H.); guisela.fernandez3@gmail.com (G.F.-E.); 3 Fundación de Oftalmología Médica de la Comunitat Valenciana, 46015 Valencia, Spain; 4 Department of Ophthalmology, Lozano Blesa University Hospital, 50009 Zaragoza, Spain; 5 Department of Ophthalmology, Miguel Servet University Hospital, 50009 Zaragoza, Spain; 6 Department of Applied Physics, University of Zaragoza, 50009 Zaragoza, Spain; 7 Department of Surgery, University of Zaragoza, 50009 Zaragoza, Spain

**Keywords:** microperimetry, MAIA, peripapillary retinal nerve fiber layer, retinal thickness, rhegmatogenous retinal detachment, optical coherence tomography, pars plana vitrectomy, swept-source-OCT, surgery

## Abstract

**Objectives:** In this study, the objective was to assess the correlation between macular function and peripapillary retinal nerve fiber layer (pRNFL) thickness following successful rhegmatogenous retinal detachment (RRD) surgery, as well as the subsequent recovery of visual acuity. **Methods**: This was a cross-sectional study including 64 eyes from patients with RRD who underwent successful treatment with 23G pars plana vitrectomy (PPV), endophotocoagulation and sulfur-hexafluoride (SF6) were included and compared to a control group consisting of 136 healthy eyes. A complete ophthalmological examination was performed on all participants, including assessment of macular sensitivity using macular integrity assessment (MAIA) microperimetry and pRNFL thickness using DRI-Triton swept-source (SS)–optical coherence tomography (OCT). **Results**: In the RRD group, retinal sensitivity was decreased. The temporal (T) sector of the total retina (TR) protocol was thicker, while the superior (S) and inferior (I) sectors of the pRNFL protocol were thinner. Within the RRD group, positive correlations were observed between the nasal (N), I sectors and total thickness of TR protocol and MAIA inferior outer (IO) sector; the I sector and total thickness of the TR protocol and MAIA inferior inner (II) sector; the I sector of the pRNFL protocol and MAIA IO sector. Negative correlations were shown between the S, T sectors and total thickness of the pRNFL protocol and MAIA central (C) sector; the N sector and total thickness of the pRNFL protocol and MAIA central temporal (CT) sector. **Conclusions**: RRD leads to a decrease in pRNFL thickness accompanied by reduced macular sensitivity. These changes may be attributed to factors such as the specific location of the RRD, the distribution pattern of the RNFL and the chosen surgical approach.

## 1. Introduction

Rhegmatogenous retinal detachment (RRD) requires rapid surgical treatment to prevent visual loss. Its incidence ranges from 9.5 to 18.2 cases per 100,000 individuals according to various studies [1].

Despite continuous technical advances and an early and anatomical successful treatment, the restoration of best corrected visual acuity (BCVA) and other visual function parameters remains unpredictable [2,3,4]. This variability has been attributed to morphological, microvascular and functional changes at the macular level, particularly at the foveal and parafoveal regions [5].

Optical coherence tomography (OCT) provides high-quality two- or three-dimensional images of the retina, choroid and optic nerve (ON), with an accurate evaluation of their anatomy and thickness [6,7]. Its speed and ease of use have made OCT a standard component of ophthalmic examinations. OCT–angiography (OCTA) further enhances diagnostic capabilities by offering detailed imaging of capillary morphology, which reveals microvascular changes in various diseases, including RRD [8,9]. In previous research, we have investigated OCTA’s functional implications using macular integrity assessment (MAIA) microperimetry [10]. MAIA allows for a detailed, point-by-point evaluation of the macula while providing real-time retinal images and information on the fixation ability [11,12].

However, the impact of RRD is not limited to the macular region, as it may affect the entire retina. Whether induced by the RRD itself or due to the surgical procedure, the retinal nerve fiber layer (RNFL) can be damaged [13,14,15].

The aim of this study is to investigate the structural ON damage in patients who have undergone RRD surgery, as assessed by OCT, and to correlate these structural changes with functional outcomes, mainly final BCVA and macular sensitivity evaluated through MAIA.

## 2. Materials and Methods

This cross-sectional, single-center study was registered and approved by the Aragon Clinical Research Committee and was conducted in accordance with the tenets of the Helsinki Declaration. It was carried out at the Department of Ophthalmology, Lozano Blesa University Hospital (Zaragoza, Spain) between July 2021 and July 2022. The RRD group included all patients who met the inclusion criteria during the recruitment period and who provided informed consent. A total of 64 eyes of 64 patients with primary RRD due to a single tear who underwent successful surgical reattachment after a single 23G pars plana vitrectomy (PPV), endophotocoagulation and sulfur-hexafluoride (SF6) tamponade performed within 4 weeks of symptom onset were included. Enrollment occurred only after complete absorption of intraocular SF6. All surgeries in the RRD group were performed by the same expert surgeon (I.P.). The control group consisted of 136 healthy eyes, including the contralateral healthy eye from the 64 RRD patients and 1 randomly selected eye from 72 healthy volunteers. All participants were adults who signed informed consent and demonstrated sufficient cooperation and fixation capacity to undergo the examinations.

Exclusion criteria encompassed pre-existing ocular or systemic conditions that could compromise BCVA or alter macular or ON anatomy. Such conditions included diabetic retinopathy (DR), any microvascular pathology affecting the posterior pole, age-related macular degeneration (AMD), pathological myopia, proliferative vitreoretinopathy above grade B, macular hole or pseudohole, epiretinal membrane (ERM), glaucoma, any neuropathy, clinically significant cataract or a previous history of RRD.

Patient demographics (age, sex and medical history) were recorded. Patients were queried regarding the onset of symptoms. The ophthalmological examination included BCVA (LogMAR), axial length (AL) using the Aladdin KR-1W (Topcon Corporation, Tokyo, Japan), intraocular pressure (IOP) measured with a Goldmann tonometer and an anterior segment examination.

**Figure 1:**
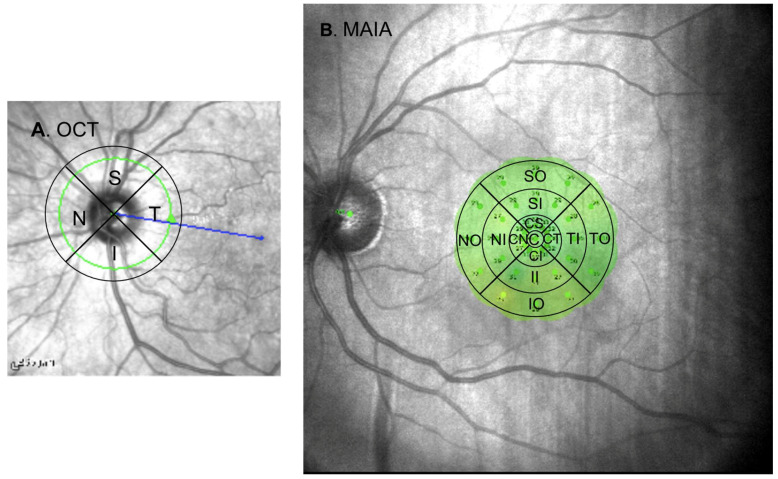
(**A**) Simplified representation of the OCT grid overlaid on a fundus image, with sector acronyms indicated in their corresponding regions. (**B**) Simplified image of the MAIA grid superimposed on a macular image as provided by the device, with the respective sector labels. Abbreviations: S: superior; T: temporal; I: inferior; N: nasal; C: central; CS: central superior; CT: central temporal; CI: central inferior; CN: central nasal; SO: superior outer; SI: superior inner; TO: temporal outer; TI: temporal inner; IO: inferior outer; II: inferior inner; NO: nasal outer; NI: nasal inner; OCT: optical coherence tomography; MAIA: macular integrity assessment.

Macular retinal sensitivity was assessed using the MAIA microperimeter (Macular Integrity Assessment system, CenterVue SpA, Padova, Italy) following an Expert Exam protocol based on a 4–2 strategy. The testing grid was divided into 3 concentric rings: central (C), inner (I) and outer (O). The latter two rings were further subdivided into 4 areas: superior (S), temporal (T), inferior (I) and nasal (N), with each sector named according to its area and corresponding ring (Figure 1).

Three stimuli were projected in each sector, and their responses were averaged. The C point along with the 12 surrounding stimuli, all included within the C circle, were analyzed as a single unit, referred to as “C global”. The macular integrity index and average total threshold were also recorded in decibels (dB). An eye tracker system quantified eye movements, allowing patient fixation to be classified based on stability parameters (“P1” and “P2”), fixation losses (expressed as percentage) and dispersal measured by the bivariate contour ellipse area (BCEA) at 63% and 95% angles (in degrees) [10]. Further details are provided in the Supplementary Materials—Figure S1.

Following pupil dilation with mydriatic drops (Tropicamide^®^, Alcon Cusi, Barcelona, Spain) fundus examination, macular and ON OCT (Deep Range Imaging (DRI)-Triton swept-source (SS)–OCT system (Topcon Corporation, Tokyo, Japan)) were performed. In the ON OCT, both total retina (TR) thickness and the peripapillary RNFL (pRNFL) protocols were analyzed. Both are automatically provided by the device. In both protocols, the grid is divided into 4 sectors—S, T, I and N. Their average is referred to as total thickness. All thickness values are in microns (Figure S2).

### Statistical Analysis

Statistical analysis was conducted using SPSS (SPSS 25, SPSS Inc., IBM Corporation, Armonk, NY, USA). Normal distribution was assessed using the Kolmogorov–Smirnov test; however, as most parameters did not follow a normal distribution, non-parametric tests were used. Differences between groups were identified using the Mann–Whitney U test for independent samples. Spearman’s correlation analysis was utilized to examine relationships between variables. The statistical significance level was set at *p* < 0.05.

## 3. Results

### 3.1. Demographics

A total of 200 eyes were analyzed, including 64 from patients in the RRD group and 136 healthy eyes in the control group. Demographic data for both groups are presented in Table 1.

### 3.2. Differences Between Groups

No significant differences were found in terms of age, sex, laterality of the studied eye, AL or IOP. Statistically significant differences between BCVA at the time of the examination between the RRD group and the control group were observed (*p* < 0.001), with lower BCVA in the RRD group.

**Figure 2:**
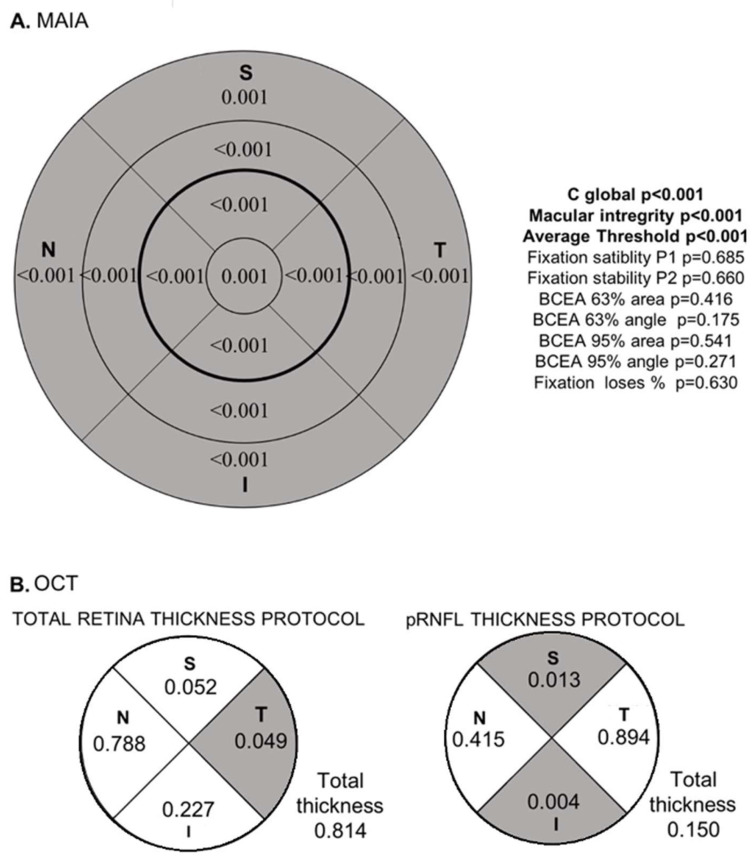
(**A**) Simplified depiction of the MAIA grid. (**B**) OCT optic nerve grid for the two studied protocols. In the grids, shaded areas indicate statistically significant differences between the RRD control groups. The corresponding *p*-values are displayed numerically. Other MAIA variables are shown in figure (**A**, **right**). Values that reached statistically significant differences are highlighted in bold. Abbreviations: MAIA: macular integrity assessment; OCT: optical coherence tomography; ON: optic nerve; pRNFL: peripapillary retinal nerve fiber layer; S, superior; I, inferior; T, temporal, N, nasal.

MAIA values are depicted in Figure 2. We observed a statistically significant decrease in sensitivity in the RRD group across all sectors. Additionally, the RRD group demonstrated a significantly lower macular average threshold value and a higher macular integrity value. There were no statistically significant differences in fixation parameters between the groups.

When analyzing the peripapillary OCT, statistically significant differences were identified in the T sector of the TR protocol, with increased thickness in the RRD group (*p* = 0.049). In the pRNFL thickness protocol, the RRD group showed lower thicknesses in the S and I sectors (*p* = 0.013, *p* = 0.004).

### 3.3. Correlations Within the RRD Group Between Peripapillary OCT and Other Variables

No significant correlations were found between ON OCT measurements and age, preoperative macular status, IOP, the time from symptom onset to the emergency room visit, or time from symptom onset to surgery.

BCVA was negatively correlated with certain OCT sectors, including total thickness and the S sector of the TR protocol, as well as the S sector of the pRNFL protocol. Patients with higher AL values exhibited a thickness reduction, showing negative correlations with the S, I and N sectors, as well as with the total thickness of both TR and pRNFL protocols. A longer interval between surgery and the examination was negatively correlated with nearly all sectors evaluated by the pRNFL protocol, except for the T sector (see Table 2).

Correlations between macular function measured by MAIA and ON OCT are presented in Table 3 and Table 4.

Macular function and ON OCT showed different correlations. Within the TR protocol, positive correlations were identified between the N OCT sector and the MAIA inferior outer (IO) sector (*p* = 0.036), the I OCT sector and MAIA IO and inferior inner (II) sectors (*p* = 0.015, *p* = 0.033), and total thickness and MAIA IO and II sectors (*p* = 0.029, *p* = 0.045). The pRNFL protocol revealed a positive correlation between the I sector and MAIA IO sector (*p* = 0.044).

In the pRNFL protocol, negative correlations were identified between the S and T OCT sectors and MAIA C sector (*p* = 0.048, *p* = 0.024); the N OCT sector and MAIA central temporal (CT) sector (*p* = 0.036); and total thickness and MAIA C and CT sectors (*p* = 0.006, *p* = 0.036).

Additional MAIA parameters exhibited diverse correlations. In the TR protocol, a positive correlation was observed between the T sector and average threshold (*p* = 0.048), and the S sectors of both TR and pRNFL protocols with fixation stability P1 (*p* = 0.041, *p* = 0.048). Negative correlations were observed between the I sector of the pRNFL protocol and macular integrity (*p* = 0.042); and in the TR protocol, between the S sector and BCEA 63% area (*p* = 0.038), and the T sector and both BCEA 63% and 95% angles (*p* = 0.33, *p* = 0.042).

### 3.4. Subgroups Analysis According to Macular Status Prior to Surgery

The RRD group was further divided into 31 macula-ON patients and 33 macula-OFF patients.

Statistically significant differences were only observed regarding MAIA parameters, specifically in the SI and CN sectors (*p* = 0.034, *p* = 0.002), C global (*p* = 0.022) and average threshold (*p* = 0.002), with higher values noted in the macula-ON group. Macular integrity showed statistically significant differences, with higher values for the macula-OFF group (*p* = 0.024).

No significant differences were detected for any other variables.

## 4. Discussion

Anatomical success following RRD repair does not necessarily translate into the recovery of BCVA or visual quality. While anatomical and functional macular changes after RRD have been extensively studied [16,17,18], changes in pRNFL thickness remain less understood. Postoperative alterations in ON coloration are common and may be related to the RRD itself or to the surgical procedure.

To properly evaluate our findings, we must consider the inclusion and exclusion criteria. The lack of significant differences between the RRD and control groups in fixation-related parameters could reflect the requirement for a minimal level of fixation capacity, cooperation and BCVA to complete the tests, thereby excluding cases with poorer post-surgical outcomes. However, statistical differences were observed in retinal sensitivity in all grid sectors assessed by MAIA, macular integrity and average threshold, findings that align with other reported results [10]. In contrast, OCT findings revealed differences only in select sectors depending on the protocol used. This variability could be related to the location and extent of the tear and subsequent RRD. Lee et al. [19] reported a decrease in RNFL thickness after an RRD compared to the corresponding area in the fellow eye. Similarly, Bonfiglio et al. [5] demonstrated a significantly lower radial peripapillary capillary plexus (RPCP) vessel density (VD) and RNFL thickness in the areas affected by primary RRD in 53 eyes compared to the corresponding area in the healthy fellow eyes. No significant differences were noted in the undetached retinal areas between the groups. However, within the RRD group, positive correlations were observed between RPCP VD and RNFL thickness in both detached (r = 0.393, *p* < 0.001) and undetached regions (r = 0.321, *p* < 0.001).

In our study, a decrease in pRNFL thickness was noted in the RRD group at the ON level in the S and I sectors compared to the control group, probably related to ganglion cell fiber loss. However, when studying the TR protocol, the RRD group had an increase in T sector thickness, which negatively correlated with MAIA parameters. The increase in thickness, which resulted in poorer macular function, may be due to fiber swelling, possibly caused by mechanical damage to the peripapillary capillaries, leading to an impaired ON fiber perfusion. This may result in extravasation and oedema as proposed by Ohashi et al. [20] in a study involving 22 patients with macular holes treated with PPV plus SF6. They observed that rim volumes significantly increased at 1 and 3 months postoperatively (*p* = 0.0008 and *p* = 0.009), returning to baseline levels after 6 months. These findings were not observed in nine patients with ERM who did not receive SF6, suggesting that gas tamponade may contribute transient alterations.

Considering the trajectory of nerve fibers from the macular to the ON, as described by Jansonius NM et al. [21], it is plausible that alterations in fibers originating from the macula could lead to functional changes detectable by MAIA. If these alterations extended into the peripapillary region, the T sector of the OCT would be expected to exhibit the strongest correlations, as it collects fibers from nearly the entire macula, particularly the N and C sectors, and portions of the S and I nasal side. Fibers from the more peripheral and temporal part of the S and I sectors reach the ON through the more temporal side of these sectors. The N sector of the OCT, however, does not capture macular fibers, making its observed correlations with MAIA difficult to explain. Two potential explanations exist; either these sectors correlate with the configuration (location and extent) of the detached retina outside the macula (which could indirectly influence macular function assessed by MAIA), or the observed changes may be influenced by other factors, such as surgery. Retinal displacement following RRD has been documented using autofluorescence imaging, showing displacement of major retinal vessels from their original positions. If nerve fibers also undergo such displacement, establishing direct equivalence between structural and functional correlations becomes challenging, particularly in the lower sectors of MAIA [22].

Bansal et al. [23] reported four cases of optic neuropathy after PPV for RRD, resulting in significant BCVA loss. Although the autoregulatory mechanisms help maintain ON head perfusion despite IOP fluctuations during surgery, their effectiveness seems to be lower in older subjects and those with local or systemic disorders such as diabetes mellitus, hypertension, hypotension, vasospasm and atherosclerosis [14,24]. Retrobulbar anesthesia reduces perfusion through both direct and indirect mechanisms [25]. The use of perfluorocarbon, different buffers, fluid–air exchange, mechanical manipulation or vigorous infusion withdrawal can cause ON damage [20,26]. Postoperative prone position could raise IOP [27]. Other factors, such as prolonged surgical time or accidental ON contact could potentially lead to neuropathy [13]. Compared to the cases described by Bansal et al. [23], our sample size is larger, and BCVA is significantly better with no cases of neuropathy after PPV. To minimize potential biases, all surgeries were performed by the same surgeon using consistent procedures and equipment. However, unnoticed factors could still contribute to RNFL damage. If the observed alterations were caused by factors other than the RRD itself, we would expect them to affect the entire posterior retina equally, unless certain areas are inherently more vulnerable than others.

Koutsandrea et al. [26] conducted a prospective study evaluating visual field (VF) defects and RNFL thickness in two groups affected by RRD treated either with scleral buckling (SB) (25 eyes) or PPV with C3F8 gas tamponade (25 eyes). They observed a decline in VF function in the PPV group compared to the SB group (−8.2 ± 5.1 vs. −5.1 ± 3.4) and a decrease in non-detached areas in the PPV group (−7.8 ± 5.1) compared to SB (−4.3 ± 3.3). They concluded that the impact of the RRD itself surpasses the effects of surgical intervention. Furthermore, they suggested that RRD and PPV may affect different retinal structures. While RRD primarily impacts the outer retina on both functional and structural levels [28], the surgical approach may contribute to additional changes. Following a similar perspective, Takkar et al. [15] analyzed 32 cases of RRD treated with PPV and silicon oil, followed by subsequent oil removal. They observed a reduction in RNFL thickness that was negatively correlated with final BCVA, mainly in the T sector (26%), followed by I (21%), S (19%) and the N sectors (18%). A significant correlation was observed between final BCVA and the timing of surgical intervention, with better visual outcomes in patients who underwent surgery sooner (r = 0.56; *p* = 0.001). These results align with previous research suggesting that the time between symptom onset and surgery, along with preoperative BCVA, are among the most reliable predictors of final BCVA [7]. Takkar et al. [15] also reported that central foveolar thickness decreased more significantly (12.5%) than ON thickness, potentially related to Müller cell proliferation and fibrosis. Their cohort exhibited worse final BCVA than our series, along with decreased retinal thickness in all sectors. In contrast, in our study, pRNFL thinning was mainly observed in vertical sectors. Our study exclusively used SF6 as tamponade, and cases with poor anatomical and functional outcomes were excluded. We identified a negative correlation between the time elapsed from surgery to testing and RNFL thickness in the S, I and N sectors, as well as total thickness in the pRNFL protocol. This correlation may reflect a progressive resolution of post-surgical alterations, such as ON edema [19], which could lead to subtle and gradual thinning over time. However, these changes do not uniformly affect all OCT protocols. Contradictory findings exist in the literature, as some studies have not found associations between anatomical changes (such as ellipsoid disruption, subretinal or intraretinal fluid presence) and functional outcomes such as BCVA in patients after RRD surgery [29].

When evaluating changes based on macular status prior to surgery, we observed significant differences not only in the CN sector but also in the SI, C global and macular integrity parameters, with poorer outcomes for the macula-OFF subgroup. These findings contras with an earlier study conducted by our group [10], which had a smaller sample size. The larger sample in the current study may have allowed for the detection of subtler differences. Although both studies required a minimum ability to perform the tests, a larger cohort may reveal more nuanced variations.

One limitation of our study is the large number of correlations analyzed. We did not apply multiple comparison correction tests, so only results with high statistical significance should be considered reliable. However, the rigorous methodology and consistency of significant correlations across multiple sectors adds credibility to our findings. Additionally, combining macula-ON and macula-OFF cases together could be controversial. A separate analysis of these subgroups could provide further insights. Moreover, comparing detached and undetached retinal areas would have been of great interest, as it could help clarify regional variations in functional impairment. Another challenge in analyzing peripapillary changes is that tests were performed at different postoperative time points, making it difficult to assess peripapillary alterations as a whole. Furthermore, our study lacks baseline (pre-surgical) measurements of pRNFL thickness and macular sensitivity. This limitation makes it difficult to determine whether observed changes are due to surgical intervention or pre-existing retinal damage. More permissive inclusion criteria and a prospective study design could potentially uncover more significant differences.

To our knowledge, this is the first study to correlate ON anatomical alterations with macular function as measured by MAIA microperimetry in RRD patients. Future research should not only focus on anatomical restoration but also on functional recovery, including subjective patient-reported outcomes and their impact on daily life. Understanding these factors could help refine surgical techniques and postoperative management strategies to optimize both structural and functional outcomes.

## 5. Conclusions

RRD leads to a reduction in macular sensitivity, accompanied by changes in pRNFL thickness, which may be attributed to both the detachment itself and the surgical intervention. Investigating these alterations can offer valuable insights into the impact of RRD location, the interval between symptom onset and surgery, and the influence of different surgical approaches. Future studies with larger sample sizes, prospective designs, and extended follow-up periods are crucial to further elucidate these correlations and their long-term implications.

## Figures and Tables

**Table 1:** Demographic data for both study groups.

		RRD GROUP		CONTROL GROUP
**Eyes analyzed (n = 200)**		64		136
		31 macula-ON	33 macula-OFF	
**Sex**	Female	21 (32.8%)		40 (29.4%)
	Male	43 (67.2%)		96 (70.6%)
**Studied eye**	Right eye	27 (42.2%)		52 (38.3%)
	Left eye	37 (57.8%)		84 (61.7%)
**Mean age (years)** **±** **SD**		59 ± 11.19 (33–84)		57 ± 10 (43–83)
**Axial length (mm)** **±** **SD**		23.35 ± 2.27 (22.01–25.40)		24.49 ± 2.79 (21.51–26.40)
**IOP (mmHg)** **±** **SD**		15 ± 2.39 (9–21)		13 ± 2.40 (9–19)
**BCVA (LogMAR) at the time of the examination**		0.25 ± 0.28 (1.0–0.0)		0.06 ± 0.12 (1.0–0.0)
**Location of the tear**	S	40 (62.5%)		
	T	19 (29.7%)		
	I	0 (0%)		
	N	5 (7.8%)		
**Time from the onset of symptoms to the first ophthalmological examination (patients)**	1 to 6 days	51 (79.69%)		
	7 to 14 days	7 (10.94%)		
	Over 15 days	6 (9.37%)		
	Medium (days)	3.00 ± 5.96 (0–30)		
**Mean time elapsed between the onset of symptoms and the patient undergoing surgery (days)**		8.00 ± 6.39 (1–35)		

Abbreviations: RRD: rhegmatogenous retinal detachment; SD: standard deviation; IOP: intraocular pressure; BCVA: best corrected visual acuity; S: superior; T: temporal; I: inferior; N: nasal.

**Table 2:** Correlations between peripapillary OCT, both total retina (TR) and peripapillary retinal nerve fiber layer (pRNFL) protocols (left column), with best corrected visual acuity (BCVA), axial length (AL) and days from surgery to examination in the RRD group. Statistically significant correlations are shown in bold and shaded. ** Statistically significant correlation at *p* < 0.01 (bilateral). * Statistically significant correlation at *p* < 0.05 (bilateral).

		BCVA	AL	Days from Surgery to Test
ON OCT TR S	Cc	−0.338 **	−0.321 **	−0.195
	Sig.	**0.006**	**0.010**	0.126
ON OCT TR T	Cc	−0.130	−0.173	0.005
	Sig.	0.306	0.172	0.969
ON OCT TR I	Cc	−0.174	−0.282 *	−0.124
	Sig.	0.169	**0.024**	0.334
ON OCT TR N	Cc	−0.114	−0.277 *	−0.058
	Sig.	0.368	**0.027**	0.652
ON OCT TR total thickness	Cc	−0.247 *	−0.282 *	−0.123
	Sig.	**0.049**	**0.024**	0.337
ON OCT pRNFL S	Cc	−0.272 *	−0.384 **	−0.293 *
	Sig.	**0.030**	**0.002**	**0.020**
ON OCT pRNFL T	Cc	0.003	−0.178	−0.076
	Sig.	0.981	0.158	0.552
ON OCT pRNFL I	Cc	−0.070	−0.311 *	−0.293 *
	Sig.	0.585	**0.012**	**0.020**
ON OCT pRNFL N	Cc	0.100	−0.315 *	−0.293 *
	Sig.	0.432	**0.011**	**0.020**
ON OCT pRNFL total thickness	Cc	−0.121	−0.399 **	−0.303 *
	Sig.	0.342	**0.001**	**0.016**

Abbreviations: ON: optic nerve; OCT: Optical Coherence Tomography; TR: total retina; pRNFL: peripapillar retinal nerve fiber layers; S: superior; T: temporal; I: inferior; N: nasal; Cc: correlation coefficient; Sig.: bilateral significance.

**Table 3:** Correlations between optic nerve OCT total retina (TR) protocol (top row), with MAIA microperimetry (left column) for the RRD group. Statistically significant correlations are highlighted in bold and shaded. * Statistically significant correlation at *p* < 0.05 (bilateral).

	ON OCT TR S Sector		ON OCT TR T Sector		ON OCT TR I Sector		ON OCT TR N Sector		ON OCT TR Total Thickness	
	Cc	Sig.	Cc	Sig.	Cc	Sig.	Cc	Sig.	Cc	Sig.
**MP SO**	−0.024	0.854	0.063	0.62	−0.002	0.99	−0.014	0.912	0.036	0.776
**MP TO**	0.028	0.827	0.166	0.191	0.076	0.549	0.08	0.527	0.094	0.458
**MP IO**	0.116	0.363	0.205	0.104	**0.304** *	**0.015**	**0.263** *	**0.036**	**0.274** *	**0.029**
**MP NO**	0.014	0.915	0.136	0.283	0.197	0.118	0.161	0.204	0.172	0.174
**MP SI**	0.112	0.377	0.15	0.236	0.152	0.23	0.104	0.413	0.172	0.174
**MP TI**	0.05	0.695	0.186	0.14	0.095	0.454	0.066	0.606	0.125	0.324
**MP II**	0.108	0.398	0.217	0.085	**0.267** *	**0.033**	0.19	0.133	**0.251** *	**0.045**
**MP NI**	0.006	0.962	0.146	0.25	0.153	0.228	0.146	0.251	0.129	0.308
**MP C**	−0.161	0.204	0.035	0.785	−0.114	0.371	−0.046	0.716	−0.075	0.558
**MP CS**	0.146	0.248	0.204	0.106	0.215	0.089	0.185	0.142	0.204	0.106
**MP CT**	−0.055	0.666	0.032	0.802	0.039	0.763	0.086	0.5	0.031	0.806
**MP CI**	0.136	0.282	0.185	0.143	0.17	0.178	0.158	0.214	0.203	0.109
**MP CN**	0.109	0.392	0.188	0.137	0.127	0.319	0.193	0.126	0.183	0.147
**MP C global**	0.104	0.413	0.206	0.103	0.168	0.184	0.153	0.229	0.182	0.151
**Macular integrity**	0.012	0.923	−0.157	0.214	−0.150	0.238	0.021	0.868	−0.104	0.413
**Average threshold**	0.112	0.377	**0.249** *	**0.048**	0.224	0.075	0.203	0.107	0.235	0.062
**Fixation stability P1**	**0.256** *	**0.041**	0.174	0.17	0.215	0.088	0.121	0.341	0.221	0.08
**Fixation stability P2**	0.208	0.099	0.123	0.332	0.123	0.333	0.091	0.473	0.172	0.173
**BCEA 63% area**	**−0.260** *	**0.038**	−0.171	0.178	−0.204	0.105	−0.124	0.328	−0.221	0.079
**BCEA 63% angle**	−0.112	0.378	**−0.268** *	**0.033**	−0.135	0.287	0.027	0.834	−0.161	0.204
**BCEA 95% area**	−0.254*	0.043	−0.157	0.215	−0.189	0.135	−0.121	0.34	−0.211	0.095
**BCEA 95% angle**	−0.128	0.315	**−0.255** *	**0.042**	−0.122	0.335	0.022	0.866	−0.160	0.205
**Fixation losses**	−0.002	0.985	−0.043	0.734	−0.040	0.754	−0.067	0.599	0.02	0.877

Abbreviations: MP: microperimetry; SO: superior outer; TO: temporal outer; IO: inferior outer; NO: nasal outer; SI: superior inner; TI: temporal inner; II: inferior inner; NI: nasal inner; C: central point; CS: superior central; CT: central temporal; CI: central inferior; CN: central nasal; C global: central global; BCEA: bivariate contour ellipse area; OCT: optical coherence tomography; ON: optic nerve; RNFL: retinal nerve fiber layers; S: superior; T: temporal; I: inferior; N: nasal; Cc: correlation coefficient; Sig.: bilateral significance.

**Table 4:** Correlations between optic nerve OCT pRNFL protocol (top row), with MAIA microperimetry (left column) for the RRD group. Statistically significant correlations are highlighted in bold and shaded. ** Statistically significant correlation at *p* < 0.01 (bilateral). * Statistically significant correlation at *p* < 0.05 (bilateral).

	ON OCT pRNFL S Sector		ON OCT pRNFL T Sector		ON OCT pRNFL I Sector		ON OCT pRNFL N Sector		ON OCT pRNFL Total Thickness	
	Cc	Sig.	Cc	Sig.	Cc	Sig.	Cc	Sig.	Cc	Sig.
**MP SO**	−0.127	0.317	−0.140	0.268	−0.073	0.564	−0.107	0.401	−0.177	0.162
**MP TO**	−0.152	0.231	−0.148	0.242	−0.048	0.706	−0.065	0.611	−0.172	0.174
**MP IO**	−0.021	0.868	0.03	0.814	0.253 *	**0.044**	−0.020	0.873	0.06	0.64
**MP NO**	−0.161	0.204	−0.067	0.598	0.102	0.425	−0.064	0.617	−0.078	0.54
**MP SI**	0	0.998	−0.045	0.722	0.109	0.392	−0.057	0.655	−0.022	0.863
**MP TI**	−0.101	0.428	−0.098	0.443	−0.006	0.963	−0.084	0.508	−0.120	0.345
**MP II**	−0.043	0.735	0.125	0.325	0.228	0.07	−0.030	0.816	0.057	0.657
**MP NI**	−0.160	0.208	0.077	0.545	0.061	0.631	−0.134	0.29	−0.083	0.515
**MP C**	−0.248 *	**0.048**	−0.282 *	**0.024**	−0.212	0.093	−0.196	0.121	−0.339 **	**0.006**
**MP CS**	−0.020	0.878	−0.078	0.538	0.074	0.56	−0.119	0.347	−0.060	0.637
**MP CT**	−0.207	0.1	−0.204	0.107	−0.123	0.332	−0.262 *	**0.036**	−0.263 *	**0.036**
**MP CI**	−0.025	0.843	0.096	0.45	0.103	0.419	−0.032	0.799	0.023	0.858
**MP CN**	−0.067	0.597	0.012	0.925	0.06	0.636	−0.013	0.92	−0.031	0.808
**MP C global**	−0.096	0.452	−0.104	0.411	0.002	0.985	−0.108	0.394	−0.123	0.331
**Macular integrity**	0.032	0.803	−0.093	0.464	−0.255 *	**0.042**	0.035	0.781	−0.041	0.746
**Average threshold**	−0.089	0.482	−0.063	0.622	0.094	0.462	−0.033	0.794	−0.067	0.601
**Fixation stability P1**	0.248 *	**0.048**	0.183	0.148	0.202	0.109	0.045	0.723	0.226	0.072
**Fixation stability P2**	0.15	0.238	0.16	0.207	0.138	0.275	−0.006	0.96	0.152	0.23
**BCEA 63% area**	−0.227	0.072	−0.188	0.137	−0.182	0.149	−0.024	0.85	−0.210	0.097
**BCEA 63% angle**	−0.040	0.753	−0.136	0.285	−0.002	0.989	−0.029	0.82	−0.059	0.642
**BCEA 95% area**	−0.218	0.084	−0.176	0.165	−0.174	0.169	−0.028	0.826	−0.201	0.111
**BCEA 95% angle**	−0.052	0.681	−0.144	0.257	−0.008	0.947	−0.052	0.685	−0.078	0.539
**Fixation losses**	0.125	0.327	0.14	0.269	0.05	0.697	0.106	0.404	0.092	0.471

Abbreviations: MP: microperimetry; SO: superior outer; TO: temporal outer; IO: inferior outer; NO: nasal outer; SI: superior inner; TI: temporal inner; II: inferior inner; NI: nasal inner; C: central point; CS: central superior; CT: central temporal; CI: central inferior; CN: central nasal; C global: central global; BCEA: bivariate contour ellipse area; OCT: optical coherence tomography; ON: optic nerve; RNFL: retinal nerve fiber layers; S: superior; T: temporal; I: inferior; N: nasal; Cc: correlation coefficient; Sig.: bilateral significance.

## Data Availability

Data are unavailable due to privacy and ethical restrictions.

## Supplementary Materials

The following supporting information can be downloaded at: https://www.mdpi.com/article/10.3390/biomedicines13040943/s1, Figure S1: example of a data collection sheet provided by MAIA microperimetry, following the “New Expert Exam” test on a healthy subject; Figure S2: optic nerve examination using DRI-Triton SS-OCT showing the total retinal thickness protocol and the thickness values on the grid.

## Abbreviations

The following abbreviations are used in this manuscript:

AL	axial length
AMD	age-related macular degeneration
BCEA	bivariate contour ellipse
BCVA	best corrected visual acuity
C	central
Cc	correlation coefficient
dB	decibels
DR	diabetic retinopathy
DRI	deep-range imaging
ERM	epiretinal membrane
I	(being the second letter of the abbreviation) inner
I	(being the first letter of the abbreviation) inferior
IC	inferior central
II	inferior inner
IO	inferior outer
IOP	intraocular pressure
LE	left eye
MAIA	macular integrity assessment
MP	microperimetry
N	nasal
n	sample size
NC	nasal central
NI	nasal inner
NO	nasal outer
O	outer
OCT	optical coherence tomography
OCTA	optical coherence tomography angiography
ON	optic nerve
PPV	pars plana vitrectomy
pRNFL	peripapillary retinal nerve fiber layer
RE	left eye
RNFL	retinal nerve fiber layer
RPCP	radial peripapillary capillary plexus
RRD	rhegmatogenous retinal detachment
S	superior
SB	scleral buckling
SC	superior central
SCP	superficial capillary plexus
SD	standard deviation
SF6	sulfur-hexafluoride
SI	superior inner
Sig.	statistical significance
SO	superior outer
SS-OCT	swept-source–optical coherence tomography
T	temporal
TC	temporal central
TI	temporal inner
TO	temporal outer
TR	total retina
VD	vessel density
VF	visual field
