# Supplementary material for: Correlation Analysis of Macular Function and Peripapillary Retinal Nerve Fiber Layer Thickness Following Successful Rhegmatogenous Retinal Detachment Surgery
Source: Biomedicines. 2025 Apr 11;13(4):943. doi: 10.3390/biomedicines13040943 (PMC12024603; doi:10.3390/biomedicines13040943)
Supplement: Supplementary file 1 [file biomedicines-13-00943-s001.zip › biomedicines-3494579-supplementary.pdf]

SUPPLEMENTARY MATERIALS

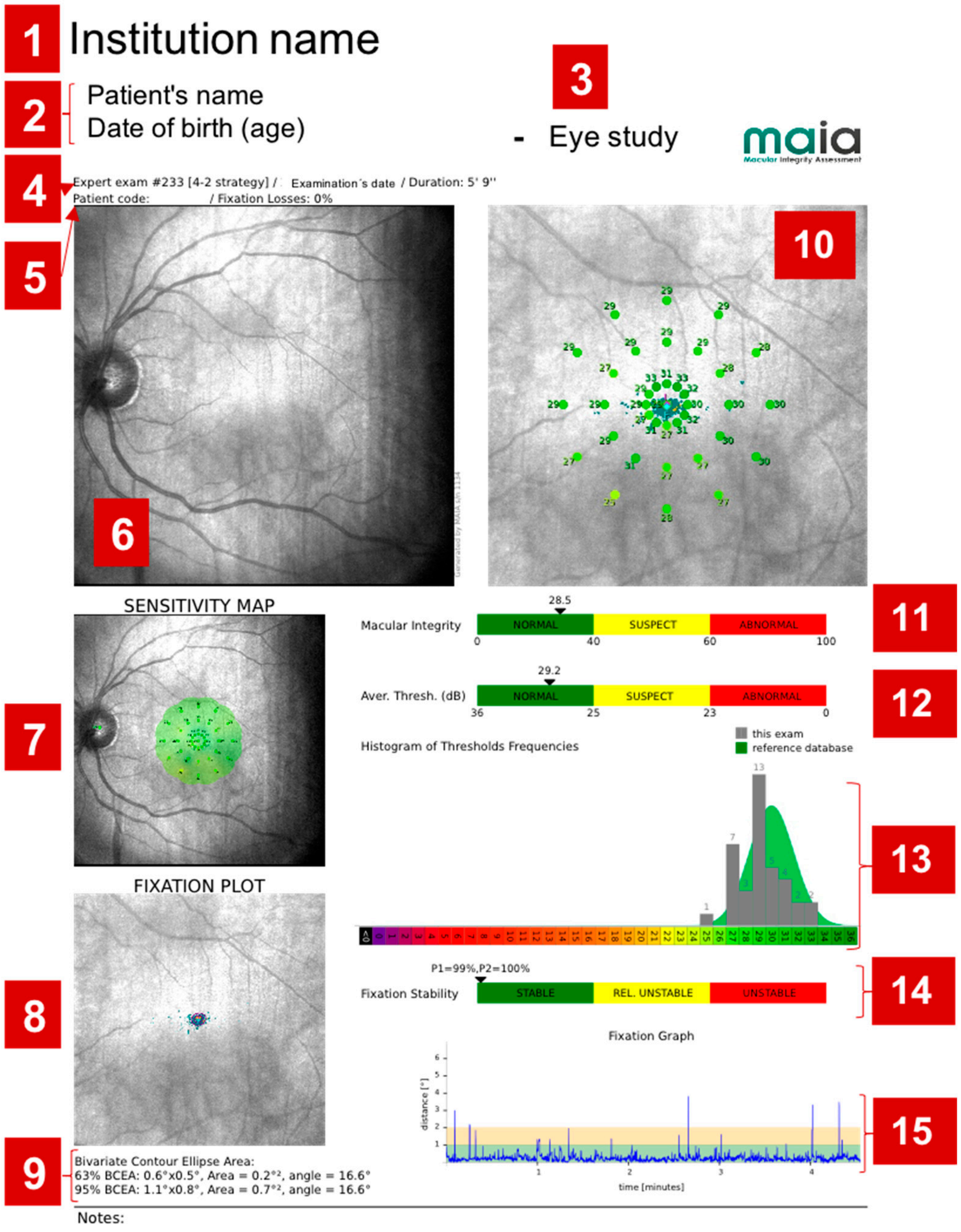

Figure S1. Example of a data collection sheet provided by MAIA microperimetry, following the "New Expert Exam" test on a healthy subject. Numbers indicate the following: **1**. Name of the institution; **2**. subject demographics; **3**. examined eye; **4**. used strategy, date, time and duration of the examination; **5**. patient code and fixation losses (%); **6**. scanning laser ophthalmoscope (SLO) fundus image; **7**. retinal sensitivity map overlapping the SLO image; **8**. fixation point cloud on the SLO fundus image and identification of the preferred retinal locus (PRL); **9**. bivariate contour ellipse area (BCEA) 63% and 95% (all fixation points recorded by the device during the examination are included in X and Y coordinates, obtaining a point cloud called BCEA with a barycenter at the final PRL); **10**. retinal sensitivity (dB) and PRL values on the SLO background image; **11**. macular integrity index (from 0 to 100 and classification according to color scale); **12**. total average threshold (from 0 to 36 dB and classification according to color scale); **13**.

histograms of threshold values (grey bars) compared to the normal distribution (green); **14**. fixation stability (P1 and P2 being P1 the percentage of points falling within a radius of 2°centred on the PRL and the percentage of points remaining within a radius of 4° centered on the PRL); **15**. fixation stability plot.

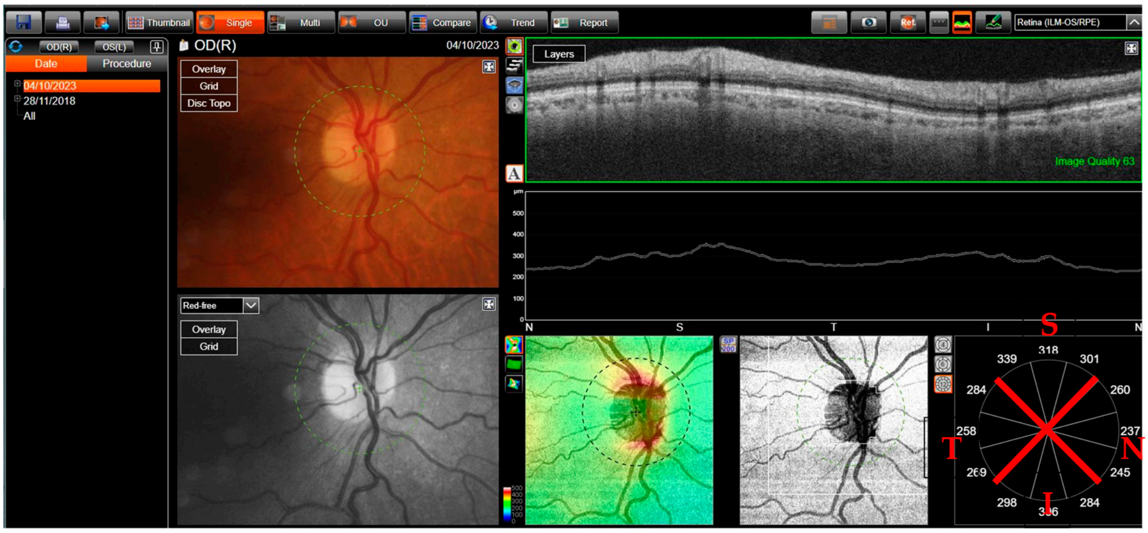

Figure S2. Optic nerve examination using DRI-Triton SS-OCT showing the total retinal thickness protocol and the thickness values on the grid. The imagen shows on the left column, the color picture and the black and white image with the examined circle. On the right column, the thickness profile, underneath the contour delimitation, and at the bottom the thickness expressed in a color-coded system, the infrared image and the thickness grid divided into 12 sectors with the thickness value expressed in microns. Three of each sectors form the four quadrants (S, superior; I, inferior; T, temporal; N, nasal) as shown with the added lines and letters.

While in the total retina (TR) protocol, the thicknesses measured correspond to the total thickness of the retina measured in the study sector, in the pRNFL protocol, only the layer that according to the automatic segmentation carried out by the device, corresponds to the nerve fiber layer would be assessed.
